# Noise and fluctuation relations of a spin diode

**DOI:** 10.1186/1556-276X-8-246

**Published:** 2013-05-20

**Authors:** Jong Soo Lim, Rosa López, David Sánchez

**Affiliations:** 1 Instituto de Física Interdisciplinar y Sistemas Complejos IFISC (UIB-CSIC), Palma de Mallorca E-07122, Spain; 2 Departament de Física, Universitat de les Illes Balears, Palma de Mallorca E-07122, Spain

**Keywords:** Spin noise, Spin diode, Fluctuation relations

## Abstract

We consider fluctuation relations between the transport coefficients of a spintronic system where magnetic interactions play a crucial role. We investigate a prototypical spintronic device - a spin-diode - which consists of an interacting resonant level coupled to two ferromagnetic electrodes. We thereby obtain the cumulant generating function for the spin transport in the sequential tunnelling regime. We demonstrate the fulfilment of the nonlinear fluctuation relations when up and down spin currents are correlated in the presence of both spin-flip processes and external magnetic fields.

## Background

Nonequilibrium fluctuation relations overcome the limitations of linear response theory and yield a complete set of relations that connect different transport coefficients out of equilibrium using higher-order response functions [1-7]. Even in the presence of symmetry-breaking fields, it is possible to derive nonlinear fluctuation relations from the microreversibility principle applied to the scattering matrix at equilibrium [5]. A possible source of time-reversal symmetry breaking are magnetized leads. Then, it is necessary to include in the general formulation the spin degree of freedom, which is an essential ingredient in spintronic applications [8] such as spin-filters [9] and spin-diodes [10-17].

We recently proved nonequilibrium fluctuation relations valid for spintronic systems [18], fully taking into account spin-polarized leads, magnetic fields, and spin-flip processes. Here, we investigate a spin diode system and explicitly demonstrate that the spintronic fluctuation relations are satisfied. Furthermore, we calculate the spin noise (correlations of the spin-polarized currents) and discuss its main properties.

## Methods

**Figure 1:**
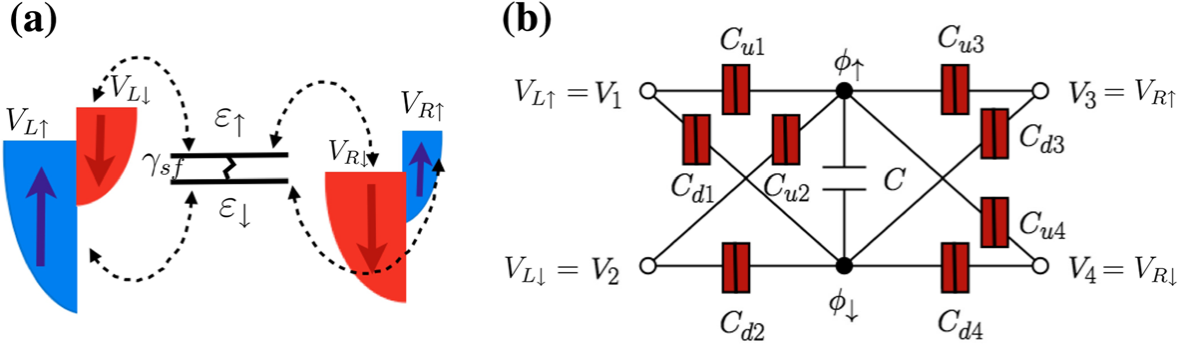
**Sketches of the spin diode system and electrostatic model.** (**a**) Sketch of the spin diode system. The dot level is attached to two ferromagnetic contacts. *V*_*Lσ*_ and *V*_*Rσ*_ indicate the spin-dependent bias voltages applied to the left (*L*) and (*R*) right contacts, respectively. The dot level is spin split by a magnetic field *B*: *ε*_*↑*_≠*ε*_*↓*_. Both spin-dependent energy levels are connected by spin-flip processes with a rate given by *γ*_sf_. (**b**) Electrostatic model: *ϕ*_*↑*_, and *ϕ*_*↓*_ are the dot internal potentials calculated using capacitance couplings [ *C*_*ui*_, *C*_*di*_ (*i*=1⋯4), *C*] within an electrostatic model.

Consider a quantum dot coupled via tunnel barriers to two ferromagnetic leads *α*=*L*,*R*, as shown in Figure 1a. The leads have spin-dependent density of states *ρ*_*α↑*(*ω*)_≠*ρ*_*α↓*(*ω*)_ (flat density of states are depicted in Figure 1a). For convenience, we introduce the leads’ spin polarization parameter as *p*_*α*_=(*ρ*_*α*_*↑*−*ρ*_*α↓*_)/(*ρ*_*α↑*_+*ρ*_*α↓*_). In the limit of $\Delta \varepsilon ≳k_{B}T,|\mathrm{eV}|$ (Δ*ε* is the dot level spacing, *k*_*B*_ is the Boltzmann constant, and *T* is the temperature) effectively only a single energy level *ε*_*σ*_ (*σ*=*↑*,*↓*) in the dot contributes to the transport and can be occupied by 0, 1, or 2 electron charges. In the presence of an external magnetic field *B*, the Zeeman splitting is *ε*_*↑*_−*ε*_*↓*_=*gμ*_*B*_*B* (*g* is the Landé factor and $\mu_{B}=\mathrm{q\hbar}/2m$ is the Bohr magneton, with *q* as the electron charge). Tunneling between lead *α* and the dot yields a level broadening given by *Γ*_*ασ*_(*ω*)=*Πρ*_*ασ*_|*V*_*α*_|^2^ (*V*_*α*_ is the lead-dot tunneling amplitude). Notice that the level width is then spin-dependent due to the spin asymmetry of the density of states: *Γ*_*ασ*_=(*Γ*/2)(1+*sp*_*α*_), with *Γ*=*Γ*_*L*_=*Γ*_*R*_ and *s*=+(−) for *↑*(*↓*).

In the limit of weak dot-lead coupling, *Γ*≪*k*_*B*_*T*, tunneling occurs sequentially, and transport is thus dominated by first-order tunnelling processes. The dynamics of the system is governed by the time evolution of the occupation probabilities calculated from the master equation dP/dt=WP, with *P*≡{*P*_0_,*P*_*↑*_,*P*_*↓*_,*P*_2_} denoting the probabilities associated to states with 0 electrons on the dot, 1 electron with spin *↑* or *↓* and 2 electrons. We also take into account spin-flip relaxation mechanisms possibly present in our system due to magnetic interactions with a spin-fluctuating environment (e.g., hyperfine coupling with nuclear spins) or spin-orbit interactions in the dot: $\gamma_{\text{sf}}^{\sigma \bar{\sigma }}=\gamma_{\text{sf}}\exp\left[\left(\varepsilon_{\sigma }-\varepsilon_{\bar{\sigma }})/(2k_{B}T\right)\right]$. To study the full counting statistics of a spin diode, we consider the generalized rate transition matrix W(χ), with *χ*={*χ*_*L↑*_,*χ*_*L↓*_,*χ*_*R↑*_,*χ*_*R↓*_} the counting fields:

(1) $$\;W(\chi )=\left(\begin{array}{cccc} -\underset{\alpha ,\sigma }{\sum }\Gamma_{\alpha \sigma }^{+} & \underset{\alpha }{\sum }\Gamma_{\mathrm{\alpha ↑}}^{-}e^{i\chi_{\mathrm{\alpha ↑}}} & \underset{\alpha }{\sum }\Gamma_{\mathrm{\alpha ↓}}^{-}e^{i\chi_{\mathrm{\alpha ↓}}} & 0\\ \underset{\alpha }{\sum }\Gamma_{\mathrm{\alpha ↑}}^{+}e^{-i\chi_{\mathrm{\alpha ↑}}} & -\underset{\alpha }{\sum }\Gamma_{\mathrm{\alpha ↑}}^{-}-\underset{\alpha }{\sum }{\tilde{\Gamma }}_{\mathrm{\alpha ↓}}^{+}-\gamma_{\text{sf}}^{\mathrm{↑↓}} & \gamma_{\text{sf}}^{\mathrm{↓↑}} & \underset{\alpha }{\sum }{\tilde{\Gamma }}_{\mathrm{\alpha ↓}}^{-}e^{i\chi_{\mathrm{\alpha ↓}}}\\ \underset{\alpha }{\sum }\Gamma_{\mathrm{\alpha ↓}}^{+}e^{-i\chi_{\mathrm{\alpha ↓}}} & \gamma_{\text{sf}}^{\mathrm{↑↓}} & -\underset{\alpha }{\sum }\Gamma_{\mathrm{\alpha ↓}}^{-}-\underset{\alpha }{\sum }{\tilde{\Gamma }}_{\mathrm{\alpha ↑}}^{+}-\gamma_{\text{sf}}^{\mathrm{↓↑}} & \underset{\alpha }{\sum }{\tilde{\Gamma }}_{\mathrm{\alpha ↑}}^{-}e^{i\chi_{\mathrm{\alpha ↑}}}\\ 0 & \underset{\alpha }{\sum }{\tilde{\Gamma }}_{\mathrm{\alpha ↓}}^{+}e^{-i\chi_{\mathrm{\alpha ↓}}} & \underset{\alpha }{\sum }{\tilde{\Gamma }}_{\mathrm{\alpha ↑}}^{+}e^{-i\chi_{\mathrm{\alpha ↑}}} & -\underset{\alpha ,\sigma }{\sum }{\tilde{\Gamma }}_{\alpha \sigma }^{-} \end{array}\right),$$

where $\Gamma_{\alpha \sigma }^{\pm }=\Gamma_{\alpha \sigma }f^{\pm }(\mu_{0\sigma }-eV_{\alpha \sigma })$, ${\tilde{\Gamma }}_{\alpha \sigma }^{\pm }=\Gamma_{\alpha \sigma }f^{\pm }(\mu_{1\sigma }-eV_{\alpha \sigma })$, and *f*^±^(*ε*)=1/[ exp(±*ε*/*k*_*B*_*T*)+1]. Here, *V*_*ασ*_ is a spin-dependent voltage bias, and *μ*_*iσ*_ is the dot electrochemical potential to be determined from the electrostatic model. *i*=0,1 is an index that takes into account the charge state of the dot. Then, the cumulant generating function in the long time limit is given by $F(\chi ;t)=\lambda_{0}(\chi )t$, where *λ*_0_(*χ*) denotes the minimum eigenvalue of W(χ) that develops adiabatically from 0 with *χ*. From the generating function, all transport cumulants are obtained [18].

We consider a gauge-invariant electrostatic model that treats interactions within a mean-field approach [19]. For the geometry sketched in Figure 1b, we employ the discrete Poisson equations for the charges *Q*_*↑*_ and *Q*_*↓*_: *Q*_*↑*_=*C*_*u*1_(*ϕ*_*↑*_−*V*_*L↑*_)+*C*_*u*2_(*ϕ*_*↑*_−*V*_*L↓*_)+*C*_*u*3_(*ϕ*_*↑*_−*V*_*R↑*_)+*C*_*u*4_(*ϕ*_*↑*_−*V*_*R↓*_)+*C*(*ϕ*_*↑*_−*ϕ*_*↓*_) and *Q*_*↓*_=*C*_*d*1_(*ϕ*_*↓*_−*V*_*L↑*_)+*C*_*d*2_(*ϕ*_*↓*_−*V*_*L↓*_)+*C*_*d*3_(*ϕ*_*↓*_−*V*_*R↑*_)+*C*_*d*4_(*ϕ*_*↓*_−*V*_*R↓*_)+*C*(*ϕ*_*↓*_−*ϕ*_*↑*_), where *C*_*ℓi*_ represent capacitance couplings for *ℓ*=*u*/*d* and *i*=1⋯4. We then find the potential energies for both spin orientations, $U_{\sigma }(N_{\sigma },N_{\bar{\sigma }})=\int_{0}^{qN_{\sigma }}dQ_{\sigma }\;\phi_{\sigma }(Q_{\bar{\sigma }},Q_{\sigma })$, *N*_*σ*_ being the excess electrons in the dot. For an empty dot, i.e., *N*_*↑*_=*N*_*↓*_=0, its electrochemical potential for the spin *↑* or *↓* level can be written as *μ*_0*σ*_=*ε*_*σ*_+*U*_*σ*_(1,0)−*U*_*σ*_(0,0). This is the energy required to add one electron into the spin *↑* or *↓* level when both spin levels are empty.

Importantly, our results are gauge invariant since they depend on potential differences ($V_{\alpha \sigma }-V_{\alpha^{\prime }\sigma^{\prime }}$) only. When the dot is charged, then *N*_*↑*_=1 or *N*_*↓*_=1, and we find $\mu_{1\sigma }=\mu_{0\sigma }+2q^{2}/\tilde{C}$, with $\tilde{C}=K/C$ and $K=\underset{i}{\sum }C_{\mathrm{ui}}\underset{j}{\sum }C_{\mathrm{dj}}+C\underset{\ell =u/d}{\sum }\underset{i}{\sum }C_{\mathrm{\ell i}}$.

## Results and discussion

### Nonlinear fluctuation relations

We denote with *α*,*β*,*γ* both the lead index and the spin channel. Thus, *α*=1 corresponds to lead *L* and spin *↑*, *α*=2 corresponds to lead *L* and spin *↓*, etc. (see Figure 1). Let *I*_*α*_ be the current operator which accounts for the spin flow in a given terminal. Then, the *I*–*V* characteristics read, up to the second order in voltage,

(2) $$\langle I_{\alpha }\rangle =\underset{\beta }{\sum }G_{\alpha ,\beta }V_{\beta }+\frac{1}{2}\underset{\beta \gamma }{\sum }G_{\alpha ,\beta \gamma }V_{\beta }V_{\gamma }\;,$$

where 〈⋯ 〉 is a quantum mechanical average. Current-current correlations (noise) between fluctuations Δ*I*=*I*−〈*I*〉 are calculated up to the first order in voltage:

(3) $$S_{\alpha \beta }\equiv \langle \Delta I_{\alpha }\Delta I_{\beta }\rangle =S_{\alpha \beta }^{\left(0\right)}+\underset{\gamma }{\sum }S_{\alpha \beta ,\gamma }V_{\gamma }\;.$$

Small fluctuations around equilibrium and their responses are related through the fluctuation-dissipation theorem. In particular, the Kubo formula for the electrical transport relates the linear conductance *G*_*α*,*β*_ (electrical response) to the equilibrium noise $S_{\alpha \beta }^{\left(0\right)}$ (equilibrium current fluctuation). Relations among the transport coefficients that appear in a nonlinear voltage expansion of the high order current cumulants have been recently obtained for spintronic systems [18]. Thus, in the weakly nonlinear transport regime we find that the equilibrium third current cumulant, $C_{\alpha \beta \gamma }^{\left(0\right)}$, is related to the second-order non-linear conductance, *G*_*α*,*βγ*_, and the noise susceptibilities, *S*_*αβ*,*γ*_, by means of a fluctuation relation,

(4) $$\begin{array}{rl} C_{\alpha \beta \gamma }^{\left(0\right)}= & k_{B}T\left(S_{\alpha \beta ,\gamma }+S_{\alpha \gamma ,\beta }+S_{\beta \gamma ,\alpha }\right)\\ & -{\left(k_{B}T\right)}^{2}\left(G_{\alpha ,\beta \gamma }+G_{\beta ,\alpha \gamma }+G_{\gamma ,\alpha \beta }\right)\;. \end{array}$$

**Figure 2:**
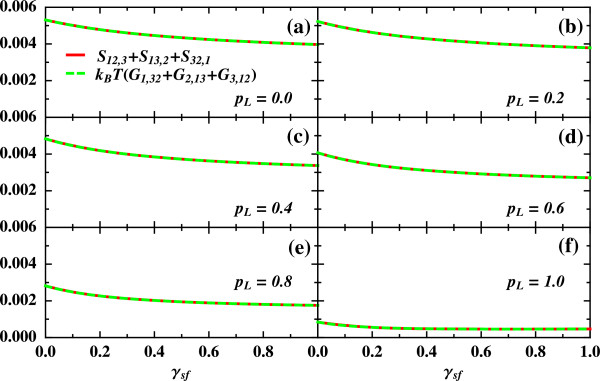
**Verification of spintronic fluctuation relations, Equation **4. Parameters: *Γ*_0_=1, *q*^2^/*C*_0_=40*Γ*_0_ (*C*_*ui*_=*C*_*di*_=*C*_0_), *C*=*∞*, *ε*_*d*_=0, *p*_*L*_=*p*≠0, *p*_*R*_=0, *k*_*B*_*T*=5*Γ*_0_, and *gμ*_*B*_*B*=0.1*Γ*_0_.

We analyze a quantum dot attached to both a ferromagnetic lead with polarization *p*_*L*_=*p* and a normal lead with polarization *p*_*R*_=0. We take into account the presence of spin-flip processes described by *γ*_sf_. In Figure 2, we explicitly check the fulfilment of Equation 4 for different values of the lead polarization in the general case of a spin-dependent bias configuration: *V*_*L↑*_=*V*_1_, *V*_*L↓*_=*V*_2_, *V*_*R↑*_=*V*_3_, *V*_*R↓*_=*V*_4_. When the dot is subjected to an externally applied magnetic field, one must consider the antisymmetrized version of Equation 4 using *A*_−_=*A*(*B*)−*A*(−*B*), where *A* can be *G*, *S*, or higher order correlation functions ($C_{-}^{\left(0\right)}=0$ for an energy-independent scattering matrix as in our system). Importantly, the checked relations involve terms of current cross correlations at different spin channels. The occurrence of nonvanishing cross correlations appears when spin-flip processes correlate the spin channels. Remarkably, only when these cross correlations are not zero, the nonlinear relations are nontrivially satisfied.

### Spin noise

We now discuss the analytical expressions for the spin noises of our spin diode. We consider that the system is biased with a source-drain voltage *V*_*SD*_=*V*_1_−*V*_3_, with *V*_1_=*V*_2_ and *V*_3_=*V*_4_. For definiteness, we take the limit *C*→*∞* (double occupation is forbidden) and zero magnetic field (*ε*_*↑*_=*ε*_*↓*_). Then, we are able to obtain an analytical expression for the cross correlations between *↑* and *↓* currents in the left terminal:

(5) $$\;S_{\;L\;↑\;L\;↓}\;\;=\;\;\left\{\begin{array}{ll} \;\;-\frac{2}{27}(1-p^{2})\Gamma_{0} & \;\text{for}\;qV_{\mathrm{SD}}>+2|\varepsilon_{\text{eff}}|\\ \;\;-\frac{2(1-p^{2})\left[(1-3p^{2})+6\gamma_{\text{sf}}/\Gamma_{0}+12{\left(\gamma_{\text{sf}}/\Gamma_{0}\right)}^{2}+8{\left(\gamma_{\text{sf}}/\Gamma_{0}\right)}^{3}\right]}{{\left(3-p^{2}\right)}^{3}+6\gamma_{\text{sf}}/\Gamma_{0}}\Gamma_{0} & \;\text{for}\;qV_{\mathrm{SD}}<-2|\varepsilon_{\text{eff}}|\;, \end{array}\right)$$

where *ε*_*eff*_=*ε*+*e*^2^/2*C*_*Σ*_, with $C_{\Sigma }=\underset{\ell ,i}{\sum }C_{\mathrm{\ell i}}$. When the level lies inside the transport window, the cross-correlations are suppressed as *p* increases independently of *γ*_sf_. Moreover, *S*_*L↑L↓*_ is always negative due to the antibunching behavior of fermions [20]. The shot noise diagonal in the spin indices is given by

(6) $$S_{\mathrm{L↑L↑}}=\left\{\begin{array}{ll} \frac{1}{27}\left(7+(5-2p)p\right)\Gamma_{0} & \;\text{for}\;qV_{\mathrm{SD}}>+2|\varepsilon_{\text{eff}}|\\ \frac{(1+p)\left[(1-p)+2\gamma_{\mathrm{sf}}/\Gamma_{0}\right]\left[(7+6p-2p^{3}+p^{4})-4(2p^{2}+p-7)\gamma_{\mathrm{sf}}/\Gamma_{0}+4(7-2p){\left(\gamma_{\mathrm{sf}}/\Gamma_{0}\right)}^{2}\right]}{{\left[(3-p^{2})+6\gamma_{\text{sf}}/\Gamma_{0}\right]}^{3}}\Gamma_{0} & \text{for}\;qV_{\mathrm{SD}}<-2|\varepsilon_{\text{eff}}|, \end{array}\right)$$

with an associated Fano factor *F*_*L↑L↑*_=*S*_*L↑L↑*_/*I*_*L↑*_,

(7) $$\;F_{\mathrm{L↑L↑}}\;=\;\left\{\begin{array}{ll} \;1-\frac{2}{9}(1+p) & \;\text{for}\;qV_{\mathrm{SD}}>+2|\varepsilon_{\text{eff}}|\\ \;1+\frac{2(1+p)\left[(4p-p^{2}-1)+2(p-2)\gamma_{\mathrm{sf}}/\Gamma_{0}-4{\left(\gamma_{\mathrm{sf}}/\Gamma_{0}\right)}^{2}\right]}{{\left[(3-p^{2})+6\gamma_{\mathrm{sf}}/\Gamma_{0}\right]}^{2}} & \text{for}\;qV_{\mathrm{SD}}<-2|\varepsilon_{\text{eff}}|. \end{array}\right)$$

Notably, the Fano factor is always sub-Poissonian whenever *ε*_*eff*_ lies inside the transport window. This is due to correlations induced by Coulomb interactions [21].

## Conclusions

Nonequilibrium fluctuation relations nicely connect nonlinear conductances with noise susceptibilities. We have derived spintronic fluctuation relations for a prototypical spintronic system: a spin diode consisting of a quantum dot attached to two ferromagnetic contacts. We have additionally investigated the fulfilment of such relations when both spin-flip processes inside the dot and an external magnetic field are present in the sample. We have also inferred exact analytical expressions for the spin noise current correlations and the Fano factor. Further extensions of our work might consider noncollinear magnetizations and energy dependent tunneling rates.

## Competing interests

The authors declare that they have no competing interests.

## Authors’ contributions

RL and DS defined the research subject. JSL and RL performed the calculations. All authors discussed the results and co-wrote the paper. All authors read and approved the final manuscript.
